# Annotations

**Published:** 1907-04-20

**Authors:** 


### ANNOTATIONS.

### Alcoholism and Insanity.

The alcohol question is emphatically one in which facts are wanted. It excites such strong feelings that to confine it within the arena of impartial scientific discussion is not an easy task. We have already expressed our opinion of the recent manifesto as an ill-advised contribution to such a discussion, and we see no reason to modify this view. As a contrast we take note of certain statements of personal experience made by Dr. Savage in the course of his Lumleian lectures. Dr. Savage recognises that alcoholic intemperance is a potent cause not only of actual insanity, but also of nervous weakness and instability, both in the individual and in his off-spring. Nevertheless, he argues that the increase of insanity at the present time certainly bears no actual relationship to the consumption of alcohol, for he feels no doubt that the English people are far more temperate than was formerly the case, and that improvement in this respect is particularly marked in the lower and middle classes. Hence the recognised increase in insanity cannot be directly placed to the charge of alcohol. Further, Dr. Savage remarks that the large number of total abstainers he sees in consulting practice has sometimes made him wonder whether "the complete and total change from moderate indulgence in alcohol to total abstinence has been altogether for the good, the mental good, I may say, of the race." But here it is necessary to remember two facts. A certain number of total abstainers adopt their position as a result of the experience of the disastrous results of excess as exhibited in their own parents, and are, by inheritance, persons of a weak and unstable nervous system. In others, total abstinence is practised as the only possible alternative to personal excess—a fact which also suggests the possession of a nervous system prone to yield to excessive strain. Such facts as these show how complex and difficult is the discussion of the whole subject of alcohol, and how necessary it is that partisan exaggeration and eloquence should be excluded from its consideration.

### The Sphygmograph.

The comparatively recent development of forms of apparatus by which the clinical estimation of the arterial blood-pressure has been reduced to a simple, and even to a merely mechanical task, has been attended by some tendency to push to one side the claims of the sphygmograph. There need be no hesitation in allowing that the modern instruments are of great value, and, indeed, they have already obtained a secure position among the methods of clinical investigation. But this by no means implies that the sphygmograph has been successfully replaced. Blood-pressure was a factor of which many physicians took due note by means of the sphygmograph long before it was clinically convenient to estimate its capacity to sustain a column of mercury, and no more exact and analytical presentation of the state of the blood-pressure can even now be recorded than is to be obtained through the agency of a sphygmographic tracing. The great advantages of the new instruments are the readiness with which they can be employed, even by the tyro, and the statement of the blood-pressure in numerical terms. The sphygmograph, on the other hand, needs a long and careful training, and this applies both to its actual employment and to the interpretation of its records; further, it does not give results capable of being expressed as those of a definite quantitative measurement. But it has the special excellence of presenting in a certain and graphic form the state of the peripheral circulation. The down stroke of the tracing is a demonstration of the ease or otherwise with which the radial artery empties itself, and is, therefore, a measure of the degree of resistance offered by the arterioles to the flow of the blood. The tracing in this way pictures the state of one of the chief factors in the determination of blood-pressure, namely, the peripheral vessels, and it separates and detaches this from other factors. In this respect it has an advantage over the sphygmomanometer, and for this reason alone it still has a large field of utility.

### Phosphorus Necrosis.

A report to the Home Secretary, by Mr. H. S. Cunynghame and Dr. B. A. Whitelegge, on the methods employed in a certain match factory, and with special reference to a recent fatal case of phosphorus necrosis, has been issued as a Parliamentary paper. The report has this satisfactory feature —namely, that it includes a statement to the effect that the instances of phosphorus necrosis recorded in the factory in question must be attributed to former, rather than to existing, conditions of employment. The former involved more exposure to phosphorus than is at present the case. Nevertheless it has to be admitted that, from the point of view of the health of the workers, even modern conditions are by no means perfect. The report in question emphasises the risk of the semi-automatic machines used in the dipping department, and suggests that it would be an improvement to dispense altogether with hand labour in this department. It, however, recognises that such a proceeding would involve a very considerable expense, and though no one likes to make the admission, the commercial bearing of this suggestion can hardly be avoided in the discussion of this question. At the same time experience has shown that when the legislative screw is applied, and when all manufacturers are made to feel its pressure, many possibilities become realised which had previously been described as impracticable. Regarding the total prohibition of the use of white phosphorus, the authors of the report above alluded to say that such a step would seem logically to involve the prohibition of the use of many other substances which have proved responsible for many more industrial accidents than can be laid to the charge of phosphorus. The alternative suggestion is the provision of completely automatic machinery, so arranged as to prevent contact between the fumes and the workers, and also between the paste and the workers' hands. It may be taken as certain that public opinion will cordially support whatever steps the Home Office may feel it necessary to take in this matter.

